# Content of Phenolic Compounds and Antioxidant Capacity in Fruits of Apricot Genotypes

**DOI:** 10.3390/molecules15096285

**Published:** 2010-09-07

**Authors:** Jiri Sochor, Ondrej Zitka, Helena Skutkova, Dusan Pavlik, Petr Babula, Boris Krska, Ales Horna, Vojtech Adam, Ivo Provaznik, Rene Kizek

**Affiliations:** 1 Department of Breeding and Propagation of Horticultural Plants, Faculty of Horticulture, Mendel University in Brno, Valticka 337, CZ-691 44 Lednice, Czech Republic; 2 Department of Chemistry and Biochemistry, Faculty of Agronomy, Mendel University in Brno, Zemedelska 1, CZ-613 00 Brno, Czech Republic; 3 Department of Biomedical Engineering, Faculty of Electrical Engineering and Communication, Brno University of Technology, Kolejni 4, CZ-612 00 Brno, Czech Republic; 4 Department of Natural Drugs, Faculty of Pharmacy, University of Veterinary and Pharmaceutical Sciences Brno, Palackeho 1-3, CZ-612 42 Brno, Czech Republic; 5 Department of Fruit Growing, Faculty of Horticulture, Mendel University in Brno, Valticka 337, CZ-691 44 Lednice, Czech Republic; 6 University Institute, Tomas Bata University in Zlin, T. G. Masaryka 275, CZ-762 72 Zlin, Czech Republic

**Keywords:** apricot, DPPH, TEAC, FRAP, high performance liquid chromatography (HPLC), electrochemical and spectrometric detection, Plum pox virus (PPV), dendrogram

## Abstract

Research on natural compounds is increasingly focused on their effects on human health. In this study, we were interested in the evaluation of nutritional value expressed as content of total phenolic compounds and antioxidant capacity of new apricot (*Prunus armeniaca *L.) genotypes resistant against Plum pox virus (PPV) cultivated on Department of Fruit Growing of Mendel University in Brno. Fruits of twenty one apricot genotypes were collected at the onset of consumption ripeness. Antioxidant capacities of the genotypes were determined spectrometrically using DPPH• (1,1-diphenyl-2-picryl-hydrazyl free radicals) scavenging test, TEAC (Trolox Equivalent Antioxidant Capacity), and FRAP (Ferric Reducing Antioxidant Power)methods. The highest antioxidant capacities were determined in the genotypes LE-3228 and LE-2527, the lowest ones in the LE-985 and LE-994 genotypes. Moreover, close correlation (r = 0.964) was determined between the TEAC and DPPH assays. Based on the antioxidant capacity and total polyphenols content, a clump analysis dendrogram of the monitored apricot genotypes was constructed. In addition, we optimized high performance liquid chromatography coupled with tandem electrochemical and spectrometric detection and determined phenolic profile consisting of the following fifteen phenolic compounds: gallic acid, 4-aminobenzoic acid, chlorogenic acid, ferulic acid, caffeic acid, procatechin, salicylic acid, *p*-coumaric acid, the flavonols quercetin and quercitrin, the flavonol glycoside rutin, resveratrol, vanillin, and the isomers epicatechin, (–)- and (+)- catechin.

## 1. Introduction

Apricots (*Prunus armeniaca* L.) were domesticated roughly 5,000 years ago in the wide area covering Iran, Turkistan, Afghanistan, Middle Asia and Western China. *Prunus armeniaca* L. is not a true native to the plains of Armenia, but it has been continuously cultivated there since at least the first century A.D. They were brought to Armenia much earlier from a more eastern centre of origin, as evidenced by archaeological excavations at pre-Christian sites. They arrived in Anatolia in the fourth century B.C. from Persia during the voyages of Alexander the Great. Thus Anatolia became the second homeland for apricots. During the Roman and Persian wars in the 1^st^ century BC, they were spread to Italy, and then to Greece. Later on, they spread to Spain and England in the 13^th^ century and to France and America in the 17^th^ century [1,2].

**Figure 1:**
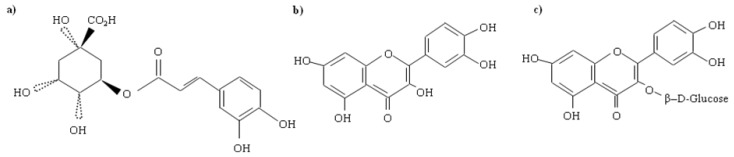
Structural formulas of (**a**) chlorogenic acid, (**b**) rutin, and (**c**) quercetin.

From a nutritional point of view, apricot pericarp contains saccharides, organic acids and mineral elements (iron, boron and potassium), vitamins such as provitamin A, vitamins B, C and polyphenols [3,4]. Richness in the above mentioned substances results in the fact that this fruit is quite widely used in folk medicine. Their anti-asthmatic and anti-anaemic effects, effects against tiredness, stress and insomnia, enhancing degradation of fats, reduction of cholesterolaemia and many others have been discussed [5,6]. It is not surprising that these beneficial effects have attracted the attention of many researchers. It was found that apricots are rich in phenolic substances, in addition to the above-mentioned compounds. Phenolic compounds, such as catechin, epicatechin, *p*-coumaric acid, caffeic acid, ferulic acid and their esters have been identified in the fruits. [7,8,9]. Chlorogenic acid (Figure 1a) is the dominant ester in apricots [5]. Flavonols occur mostly as glycosides and rutinosides of quercetin (Figure 1b), however, kaempferol and quercetin 3-rutinoside (Figure 1c) predominate [3].

Natural phenolic compounds have and continue to attract the interest of numerous scientists due to possible relations between their content in diet and lower incidence of cancer or cardiovascular diseases [10,11]. Their antimutagenic, anticarcinogenic and antiinflammatory effects have been also confirmed, but large clinical studies to support these effects in human are still lacking. In addition, some epidemiological studies indicate a connection between lower occurrence of neurodegenerative diseases and consumption of fruits and vegetables [8,12,13,14]. Besides their health effects, antioxidant activity is often discussed, because they are able to scavenge highly reactive oxygen species (ROS) damaging cell compartments [13,15,16]. It was shown that some phenolic compounds demonstrate higher antioxidant activity in comparison to others [16]. This fact is important during fruit ripening, when the contents of single phenolic compounds significantly vary. Due to the chemical variability of compounds with antioxidant capacity present in fruits, the content of certain compounds is not usually known. Determination of total antioxidant capacity is one of the ways of expressing the biological and nutritional value of fruits [15,17,18], however, the content of certain or total phenolic compounds may not correspond to the total antioxidant capacity of a fruit, because the presence of other biologically active substances including low molecular mass thiols such as glutathione participating in redox metabolism [15,16,17]. Based on the beneficial effects and economic importance of apricot fruits, we focused our attention on the nutritional characterization of twenty one new apricot genotypes (LE-3239, LE-985, LE-2527, LE-2267, LE-8175, LE-9299, LE-2927, LE-3190, LE-3276, LE-3247, LE-10278, LE-3204, LE-3241, LE-3255, LE-8561, LE-1402, LE-3228, LE-806, LE-994, LE-3187 and LE-3709) resistant against plum pox virus (PPV). Particularly, we determined total polyphenols content and antioxidant capacity using three independent assays: the DPPH• (1,1-diphenyl-2 picrylhydrazyl free radicals) test, TEAC (Trolox Equivalent Antioxidant Capacity), and FRAP (Ferric Reducing Antioxidant Power) and the chromatographic profile of phenolic compounds. In order to find markers for selection proposes, we investigated the correlations among the studied parameters.

## 2. Results and Discussion

More than 5,000 natural compounds with chemopreventive effect, especially phenolics and polyphenolics, have been identified in plants [15,19]. Their synergic effects are well known, thus the total content of these substances is of interest and can reveal important information about the nutritional values of fruits.

### 2.1. Total polyphenols content in apricot fruits

Most of phenolic compounds occurring in fruits exhibit antioxidant activity [13,20], which is defined as the ability of a compound or a mixture thereof to inhibit oxidative degradation of various substances via scavenging of reactive species, including free radicals [9,16].More than 20 methods are known and used for determination of antioxidant activity. In this study, we determined content of total polyphenols in twenty one apricot genotypes (LE-3239, LE-985, LE-2527, LE-2267, LE-8175, LE-9299, LE-2927, LE-3190, LE-3276, LE-3247, LE-10278, LE-3204, LE-3241, LE-3255, LE-8561, LE-1402, LE-3228, LE-806, LE-994, LE-3187 and LE-3709) using Folin-Ciocalteu reagent method, which is based on an oxidation-reduction reaction, in which phenolic compounds are oxidized with simultaneous reduction of phosphotungsten-phosphomolybdate complex in alkaline medium giving a blue coloration. Quality parameters such as total soluble solids (ranging from 8 to 12 °Brix), titratable acidity (ranging from 0.7 to 1.1 %) and pH (ranging from 4.0 to 5.1) of the apricot genotypes were determined too (for more details see the Experimental section). Determination of levels of total polyphenols were re-calculated based on gallic acid (GA), which varied from 41 to 170 mg GA·100 g^-1^of fresh weight (FW) and provides information about the quantitative phenolic content in apricots (Table 1). The highest values of total polyphenols were determined in genotypes LE-3228 (170 mg GA·100 g^-1^ FW) and LE‑2527 (96 mg GA·100 g^-1^ FW), and the lowest levels in genotypes LE-985 (41 mg GA·100 g^-1^ FW) and LE-994 (43 mg GA·100 g^-1^ FW). Even the lowest levels still represent considerable amounts of polyphenols. Thus, a study of their antioxidant activity followed.

**Table 1:** Antioxidant activities of the apricots determined by DPPH test, TEAC method and FRAP method re-calculated to mmol·L^-1^ of Trolox equivalent, and total polyphenols content (TP) re-calculated to equivalent of gallic acid (mg GA·100 g^-1^ FW). The samples were appropriately diluted with methanol prior to measurement of DPPH and FRAP. Data are mean of three replications ± standard deviation.

Genotype	DPPH (mmol·L^-1^)	DPPH (mg Trolox·100 g^-1 ^FW)	TEAC (mmol·L^-1^)	TEAC (mg Trolox·100 g^-1 ^FW)	FRAP (mmol·L^-1^)	FRAP (mg Trolox·100 g^-1 ^FW)	TP (mg GA·100 g^-1^ FW)
LE-806	0.80 ± 0.10	60.1 ± 5.2	0.21 ± 0.02	15.8 ± 1.1	0.38 ± 0.03	28.5 ± 3.0	67 ± 7
LE-985	0.12 ± 0.02	9.0 ± 0.8	0.04 ± 0.01	3.0 ± 0.2	0.16 ± 0.02	12.0 ± 0.9	41 ± 5
LE-994	0.25 ± 0.02	18.8 ± 1.2	0.02 ± 0.00	1.5 ± 0.1	0.22 ± 0.02	16.5 ± 1.3	43 ± 6
LE-1402	0.28 ± 0.02	21.0 ± 1.7	0.06 ± 0.01	4.5 ± 0.3	0.23 ± 0.02	17.3 ± 1.4	47 ± 5
LE-2267	0.60 ± 0.10	45.0 ± 3.9	0.08 ± 0.01	6.0 ± 0.4	0.53 ± 0.05	39.8 ± 3.3	50 ± 3
LE-2527	3.80 ± 0.40	285.3 ± 32.7	0.46 ± 0.03	34.5 ± 2.9	1.37 ± 0.09	102.9 ± 14.8	96 ± 9
LE-2927	0.25 ± 0.02	18.8 ± 1.9	0.03 ± 0.00	2.3 ± 0.3	0.25 ± 0.02	18.8 ± 1.3	46 ± 3
LE-3187	1.50 ± 0.10	11.3 ± 0.9	0.24 ± 0.02	1.8 ± 0.2	0.55 ± 0.06	41.3 ± 3.4	61 ± 5
LE-3190	0.30 ± 0.04	22.5 ± 2.1	0.05 ± 0.01	3.8 ± 0.4	0.29 ± 0.03	21.8 ± 2.0	52 ± 6
LE-3204	1.20 ± 0.20	90.1 ± 8.7	0.31 ± 0.05	23.3 ± 2.1	0.46 ± 0.04	34.5 ± 3.5	75 ± 7
LE-3228	7.40 ± 1.00	555.6 ± 48.6	1.90 ± 0.20	142.6 ± 19.7	0.22 ± 0.02	16.5 ± 1.1	170 ± 20
LE-3239	0.7 ± 0.04	52.6 ± 5.4	0.10 ± 0.02	7.5 ± 0.8	0.67 ± 0.04	50.3 ± 4.2	61 ± 6
LE-3241	0.20 ± 0.02	15.0 ± 1.2	0.03 ± 0.00	2.3 ± 0.2	0.21 ± 0.02	15.8 ± 1.1	44 ± 6
LE-3247	0.25 ± 0.03	18.8 ± 1.4	0.04 ± 0.01	3.0 ± 0.2	0.79 ± 0.04	59.3 ± 4.9	47 ± 4
LE-3255	0.24 ± 0.02	18.0 ± 1.6	0.03 ± 0.01	2.3 ± 0.2	0.53 ± 0.07	39.8 ± 3.8	51 ± 2
LE-3276	1.60 ± 0.20	120.1 ± 9.9	0.36 ± 0.06	27.0 ± 1.9	0.45 ± 0.03	33.8 ± 2.8	85 ± 8
LE-3709	0.33 ± 0.04	24.8 ± 3.1	0.06 ± 0.01	4.5 ± 0.3	0.41 ± 0.03	30.8 ± 2.5	45 ± 6
LE-8175	0.40 ± 0.04	30.0 ± 2.6	0.05 ± 0.01	3.8 ± 0.3	0.49 ± 0.02	36.8 ± 2.6	53 ± 5
LE-8561	0.36 ± 0.02	27.0 ± 2.0	0.06 ± 0.01	4.5 ± 0.3	0.35 ± 0.02	26.3 ± 2.1	52 ± 6
LE-9299	1.10 ± 0.10	82.6 ± 6.6	0.25 ± 0.01	18.8 ± 2.2	1.03 ± 0.08	77.3 ± 8.85	72 ± 5
LE-10278	0.11 ± 0.02	8.3 ± 0.7	0.03 ± 0.00	2.3 ± 0.2	0.53 ± 0.02	39.8 ± 3.1	48 ± 4

### 2.2. Antioxidant capacity in apricot fruits

To determine antioxidant activity in given biological material, it is necessary to choose an appropriate method. In spite of the fact that one only method is obviously utilized for this purpose, to ensure the objectivity of the results as well as to compare the individual techniques, we employed three methods called DPPH, TEAC and FRAP. DPPH is a method based on the ability of antioxidants to interact with stable 1,1-diphenyl-2 picrylhydrazyl (DPPH•) free radicals. The TEAC method determines the concentration of 6-hydroxy-2,5,7,8-tetramethylchroman-2-carboxylic acid (well known as Trolox) corresponding to the antioxidant activity of a sample. The FRAP technique follows the ferric to ferrous reduction of a complex catalyzed by antioxidants. Each method was calibrated on Trolox. The calibration curves of dependence of absorbance (increase/decrease) on concentration of Trolox were measured and were strictly linear in the particular concentration intervals; for more details see the Experimental section. Relative antioxidant activity was expressed as percentage of the absorbance decrease and subsequently calculated as an equivalent content of Trolox. 

The DPPH^•^ test is based on the ability of stable free radicals of 1,1-diphenyl-2 picrylhydrazyl to react with hydrogen donors. This assay is more selective compared to ABTS^•+^. In this assay, after reduction with an antioxidant (AH) or radical (R^•^), the solution is decolorized according to the following reaction: DPPH^•^+AH → DPPH‑H+A^•^, DPPH^•^+R• → DPPH‑R [21]. Antioxidant activity measured using DPPH• test in the genotypes of apricot varied within the interval from 0.11 to 3.79 mmol·L^-1^after calculation based on Trolox (Table 1). The highest antioxidant activity was determined in genotypes LE-3228 (7.4 mmol·L^-1^), LE-2527 (3.79 mmol·L^-1^) and LE-3276 (1.64 mmol·L^-1^), and the lowest in LE-10278 (0.11 mmol·L^-1^) and LE-985 (0.12 mmol·L^-1^).

The TEAC method (Trolox Equivalent Antioxidant Capacity) is one of the most used methods for quantifying radicals which can be scavenged by some antioxidant [13]. It is based on scavenging of the cation radical originated by the one-electron oxidation of the synthetic chromophore 2,2‘-azinobis(3-ethylbenzothiazoline-6-sulfonate(ABTS^•^) to ABTS^•+^. This reaction is monitored photometrically based on the change of the corresponding absorption spectrum. For pure compounds, TEAC is defined as the micromolar concentration of Trolox equivalents demonstrating the same antioxidant activity as the tested compound (at a concentration of 1 mmol·L^-1^) [16,18]. Genotypes of apricot evaluated by us demonstrated four times lower values (0.04–1.90 mmol·L^-1^ Trolox equivalents, Table 1) in comparison with the above described DPPH• test. This fact relates with the different reaction mechanisms of both methods. The highest antioxidant activity was determined in genotypes LE-3228 (1.90 mmol·L^-1^),LE-2527 (0.46 mmol·L^-1^) and LE-3276 (0.36 mmol·L^-1^), and the lowest in LE-10278 (0.03 mmol·L^-1^), LE-3241 (0.03 mmol·L^-1^) and LE-3255 (0.03 mmol·L^-1^). These results are in very good agreement with the DPPH test ones.

Antioxidant activities of the apricots, which were detected using the FRAP method − [4], varied within the range from 0.16 to 1.3 mmol·L^-1^ of Trolox equivalents. The method itself is based on the reduction of ferric complexes, e.g. 2,4,6-tripyridyl-*s*-triazine (TPTZ) with potassium ferricyanide K_3_[Fe(CN)_6_] or ferric chloride FeCl_3_, which are almost colourless or slightly brownish. After reduction, a blue-coloured ferrous complex is formed [22]. The results are shown in Table 1. The highest antioxidant activity according to FRAP method was determined in genotypeLE-2527 (1.37 mmol·L^-1^) and LE-9299 (1.03 mmol·L^-1^), and the lowest in LE-985 (0.16 mmol·L^-1^) and LE-3241 (0.21 mmol·L^-1^). This method has limitations including the low pH (pH = 3.6) of the measured samples. Another limitation is that some compounds, such as slowly reacting polyphenols and thiols, are not detected. The FRAP method only reflects the ability of compounds to reduce Fe(III) ions, which may not correlate with the true antioxidant activity of a sample [22,23].

**Figure 2:**
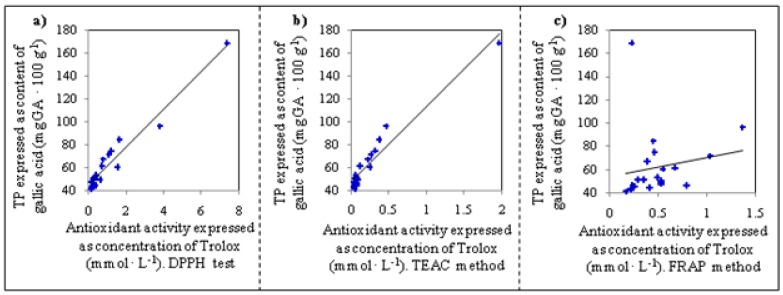
Correlation between total polyphenol content (TP) and single methods used for determination of antioxidant activity of apricots. (**a**) DPPH test *vs.* TP, (**b**) TEAC method *vs.* TP, (**c**) FRAP method *vs.* TP.

Further, we were interested in the issue of whether the methods used for characterization of the apricots correlate. We found a high positive correlation (r = 0.964) between the TEAC and DPPH assays. Correlation of the FRAP method with the other methods were positive too, but very low (r = 0.378 with DPPH test and r = 0.439 with TEAC). However, the correlation between methods used and total content of polyphenols (TP) was the most important. The obtained results are shown in Figure 2. For the DPPH test and TEAC method, highly positive correlations with TP were determined (DPPH *vs.* TP r = 0.972 and TEAC *vs.* TP r = 0.967). Nevertheless, the correlation of the FRAP method with TP was relatively low (FRAP *vs.* TP r = 0.278), which can be associated with the fact that this method does not detect all compounds possessing antioxidant activity.

### 2.3. Chromatographic analysis

**Figure 3:**
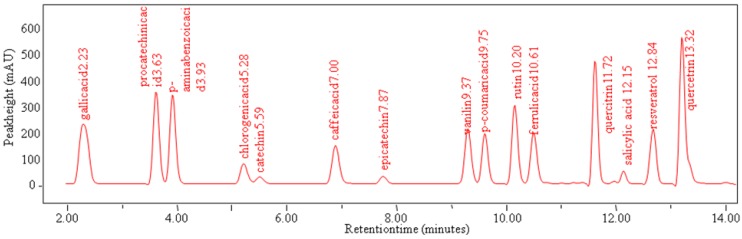
Chromatogram of standards phenolic compounds (retention times in minutes for gallic acid 2.23; procatechin 3.63; 4-aminobenzoic acid 3.93; chlorogenic acid 5.28; catechin 5.59; caffeic acid 7.00; epicatechin 7.87; vanillin 9.37; *p*-coumaric acid 9.75; rutin 10.20; ferulic acid 10.61; quercitrin 11.72; salicylic acid 12.15; resveratrol 12.84; quercetin 13.32).

Based on the above-mentioned results it can be concluded that antioxidant activity is closely correlated with the total content of polyphenols. Therefore, we were further interested in the more detailed characterization of the apricots. We aimed our attention on the following fifteen phenolic compounds: gallic acid, 4-aminobenzoic acid, chlorogenic acid, ferulic acid, caffeic acid, procatechin, salicylic acid, *p*-coumaric acid, the flavonols quercetin and quercitrin, the flavonol glycoside rutin, resveratrol, vanillin, and the isomers epicatechin, (–)- and (+)- catechin. These compounds are also interesting due to their health benefits. Fruits of apricots of given genotypes were collected at consumption ripeness from the 1^st^ July to the 20^th^ August. Fruits were homogenized and phenolic compounds subsequently extracted using very simple process described in the Experimental section. A chromatographic method with multi-step linearly increasing gradient for rapid and effective separation of the 15 phenolic compounds has been optimized. Time of the analysis of one sample including regeneration of the chromatographic column was only 25 minutes. The chromatogram obtained is shown in Figure 3. The contents of all target molecules are shown in Table 2. Chlorogenic acid is one of the principal components, especially in members of genus *Prunus* [13]. Average content of this acid in the twenty one genotype of apricots varied from 121 to 944 mg·100 g^-1^. Derivatives of hydroxybenzoic acid are present in fruits, especially in the form of esters, as gallic acid and salicylic acid [12,15,18]. Gallic acid was of the most abundant among these compounds (the quantity varied from 1 to 66 mg·100 g^-1^). From the group of antioxidants with a flavonoid skeleton, the flavonol quercetin was the most abundant compound (from 2 to 73 mg·100 g^-1^). Contents of the other flavonoid, the flavonol glycoside rutin, was within the range from 1 to 33 mg·100 g^‑1^. Moreover, phenolic compounds are very suitable for utilization as chemotaxonomic markers. Qualitative and quantitative composition of phenolics in fruits is typical and unique for individual species and genotypes [15,17] and can be used fordividing their chemotaxonomy.

### 2.4. Plum pox virus

Plum pox virus (PPV) belongs to the group of five most dangerous viral diseases of fruit [22,24]. The economic impact of viral disease consists in a lowering of the commercial value of the production, because the disease significantly reduces yields and quality of apricots. PPV can be eliminated in individual species of fruit using classical *in vivo* thermotherapy or chemotherapy [23,25]. Only very few papers focused on sanitation of fruit species from PPV have been published, however, the procedures are different, ambiguous and generally unreproducible [23,25,26,27]. Therefore, it is highly desirable to find ways to achieve relative resistance by experiments aimed at resistance against this virus. In this study, we attempted to find a correlation between antioxidant capacity and total phenolic compounds content of the single apricot genotypes and their resistance against PPV. From the set of genotypes, seven of them were included in the category of “resistant”, eight in “moderately resistant” and six in “vulnerable”. The results of this evaluation were related to the results of determining antioxidant activity and levels of total phenolic compounds content. Correlation coefficients with the methods of antioxidant activity (DPPH r = 0.351, r = 0.256 TEAC, FRAP r = 0.123) and with the content of polyphenols (TP r = 0.152) were very low. The obtained results suggest that the antioxidant capacity and total phenolic compounds content are not directly associated with resistance to PPV.

**Table 2:** Content of target phenolic compounds (GA-gallic acid; PRA-procatechin (acid); 4A-4aminobenzoic acid; CH-chlorogenic acid; CT-catechin; CA-caffeic acid; EP-epicatechin; VA-vanillin; *P*CA-*p*-coumaric acid; RU-rutin; FA-ferulic acid; QI-quercitrin; SA-salicylic acid; RE-resveratrol; QE-quercetin) in single genotype. All values are given as mg·100 g^-1^ of fresh weight. ND – not detected. Data are mean of three replications ± standard deviation.

	GA	PRA	4-A	CH	CT	CA	EP	VA	PCA	RU	FA	SA	RE	QE
time (min.)	2.23	3.63	3.93	5.28	5.59	7.00	7.87	9.37	9.75	10.20	10.60	12.15	12.80	13.30
LE-806	4.3 ± 0.5	4.2 ± 0.5	19 ± 2	790 ± 40	5 ± 1	1.1 ± 0.1	1.8 ± 0.2	2.3 ± 0.2	2.7 ± 0.2	2 ± 1	0.32 ± 0.07	19 ± 2	0.68 ± 0.07	8 ± 1
LE-985	0.4 ± 0.1	0.5 ± 0.1	2.7 ± 0.3	160 ± 20	7 ± 1	0.5 ± 0.1	0.2 ± 0.1	0.6 ± 0.1	0.4 ± 0.1	9 ± 1	0.26 ± 0.05	4 ± 1	0.45 ± 0.05	2 ± 1
LE-994	1.3 ± 0.2	2.9 ± 0.4	6.0 ± 0.5	160 ± 10	5 ± 1	0.5 ± 0.1	2.6 ± 0.2	1.2 ± 0.1	1.1 ± 0.1	11 ± 2	0.10 ± 0.02	5 ± 1	0.70 ± 0.07	32 ± 3
LE-1402	0.2 ± 0.1	2.3 ± 0.3	6.4 ± 0.5	230 ± 20	2 ± 1	0.4 ± 0.1	1.4 ± 0.2	0.6 ± 0.1	0.5 ± 0.1	2 ± 1	0.09 ± 0.03	8 ± 1	0.53 ± 0.05	73 ± 6
LE-2267	0.2 ± 0.1	0.5 ± 0.1	ND	860 ± 40	4 ± 1	0.6 ± 0.1	0.7 ± 0.1	1 ± 0.1	1.2 ± 0.1	5 ± 1	0.19 ± 0.02	3 ± 1	0.03 ± 0.01	19 ± 3
LE-2527	6.7 ± 0.4	43 ± 6	7.7 ± 0.5	120 ± 10	54 ± 5	7.3 ± 0.3	11 ± 0.9	8.3 ± 0.6	8.8 ± 0.6	25 ± 3	0.51 ± 0.05	13 ± 2	0.08 ± 0.02	16 ± 2
LE-2927	4.6 ± 0.5	2.7 ± 0.3	13 ± 0.1	930 ± 40	2 ± 1	0.5 ± 0.1	2.3 ± 0.2	1.4 ± 0.1	3.6 ± 0.4	5 ± 1	1.32 ± 0.09	23 ± 2	0.24 ± 0.03	6 ± 1
LE-3187	0.7 ± 0.1	17 ± 2	7.3 ± 0.4	630 ± 30	12 ± 1	0.7 ± 0.1	4.4 ± 0.3	1.2 ± 0.1	0.6 ± 0.1	11 ± 2	0.12 ± 0.02	73 ± 6	1.31 ± 0.09	11 ± 1
LE-3190	5.9 ± 0.4	2.5 ± 0.3	4.1 ± 0.4	410 ± 30	6 ± 1	ND	4.5 ± 0.4	1.4 ± 0.1	1.5 ± 0.1	2 ± 1	0.62 ± 0.04	11 ± 1	0.34 ± 0.03	8 ± 1
LE-3204	1.2 ± 0.1	28 ± 5	8.5 ± 0.7	940 ± 50	13 ± 1	0.6 ± 0.1	2.4 ± 0.4	0.8 ± 0.1	1 ± 0.1	33 ± 5	0.10 ± 0.02	4 ± 1	0.29 ± 0.03	47 ± 5
LE-3228	0.3 ± 0.1	4.6 ± 0.5	ND	480 ± 30	21 ± 2	0.2 ± 0.1	3.6 ± 0.4	1.5 ± 0.1	1.5 ± 0.1	8 ± 1	0.49 ± 0.06	7 ± 1	0.59 ± 0.06	4 ± 1
LE-3239	3.6 ± 0.5	22 ± 2	5.2 ± 0.4	140 ± 10	30 ± 2	3.9 ± 0.4	5.7 ± 0.4	4.4 ± 0.3	4.6 ± 0.4	17 ± 2	0.39 ± 0.07	9 ± 1	0.26 ± 0.04	9 ± 2
LE-3241	2.7 ± 0.4	29 ± 4	6.3 ± 0.4	150 ± 10	23 ± 2	2.9 ± 0.2	9.5 ± 0.6	1.2 ± 0.1	0.6 ± 0.1	9 ± 1	1.69 ± 0.09	13 ± 2	0.37 ± 0.03	17 ± 1
LE-3247	2.9 ± 0.2	3.9 ± 0.6	11 ± 1	300 ± 20	6 ± 1	0.2 ± 0.1	3.8 ± 0.3	4.5 ± 0.3	4.8 ± 0.5	8 ± 1	0.40 ± 0.04	16 ± 2	0.12 ± 0.01	10 ± 1
LE-3255	2.0 ± 0.3	10 ± 1	16 ± 2	500 ± 30	10 ± 1	1.6 ± 0.2	4.4 ± 0.3	2.7 ± 0.2	1.6 ± 0.2	6 ± 1	0.34 ± 0.04	14 ± 2	0.47 ± 0.04	40 ± 2
LE-3276	3.7 ± 0.1	3.2 ± 0.3	8.9 ± 0.8	530 ± 40	13 ± 1	5.6 ± 0.4	8.3 ± 0.7	1.9 ± 0.2	2.1 ± 0.2	8 ± 1	0.97 ± 0.06	19 ± 2	0.29 ± 0.02	23 ± 4
LE-3709	1.7 ± 0.2	2.8 ± 0.3	3.1 ± 0.4	330 ± 20	5 ± 1	0.7 ± 0.1	2.0 ± 0.1	1.1 ± 0.1	2.2 ± 0.2	6 ± 1	0.24 ± 0.02	38 ± 5	0.72 ± 0.06	4 ± 1
LE-8175	4.0 ± 0.5	2.8 ± 0.2	5.5 ± 0.3	290 ± 20	6 ± 1	0.7 ± 0.1	7.7 ± 0.6	1.4 ± 0.1	1.2 ± 0.1	9 ± 1	0.82 ± 0.08	8 ± 1	0.54 ± 0.07	23 ± 2
LE-8561	1.7 ± 0.2	35 ± 4	12 ± 1	180 ± 20	10 ± 1	3.3 ± 0.3	3.4 ± 0.4	2.5 ± 0.3	3.2 ± 0.2	14 ± 1	0.86 ± 0.07	33 ± 3	0.22 ± 0.02	7 ± 1
LE-9299	3.7 ± 0.3	22 ± 0.3	12 ± 0.2	940 ± 50	15 ± 2	0.9 ± 0.1	0.3 ± 0.1	2.7 ± 0.2	8.7 ± 0.7	6 ± 1	0.26 ± 0.02	41 ± 4	0.12 ± 0.02	19 ± 2
LE-10278	4.2 ± 0.3	6.8 ± 0.8	8.7 ± 0.6	770 ± 40	29 ± 4	1.8 ± 0.2	10 ± 1	10 ± 1	12 ± 1	34 ± 4	0.79 ± 0.03	75 ± 7	1.09 ± 0.09	35 ± 4

### 2.5. Bioinformatics analysis

**Figure 4:**
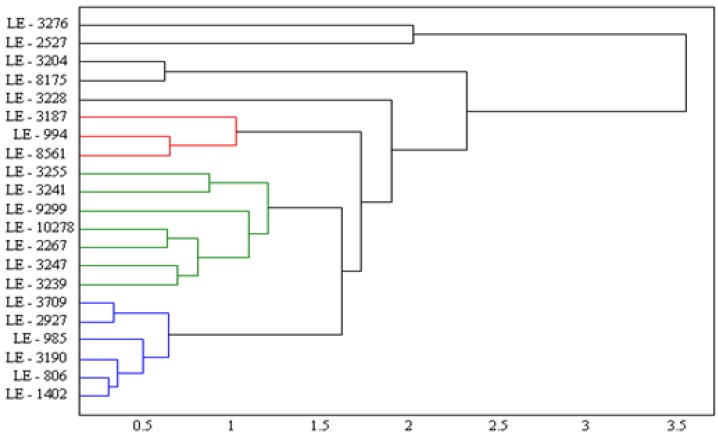
Dendrogram of clump analysis proposed on basis of Ward´s method.

Due to the broad set of the experimental data obtained, mathematical analysis was used for their interpretation. We used clump analysis for their treatment. The statistical toolbox in the program Matlab 7.9.0 (R2009b) was used for construction of the dendrogram. The clump analysis dendrogram of the monitored 21 apricot genotypes was constructed on the basis of values of antioxidant activities obtained by measurements using three different methods (FRAP, TEAC, DPPH test) with the aim of determining if this set of genotypes can be divided into separated sub-sets, which are internally homogenous, but mutually heterogeneous (Figure 4). FRAP assay did not give similar results to those measured by FRAP and TEAC. On the other hand, this assay is commonly used and is sensitive against other compounds, most probably, with altered antioxidant activity. Nevertheless, these compounds are part of a sample and, thus, their content somewhat belongs to the characterization of a fruit sample. Therefore, we included this assay to prepare the dendrogram. It follows from obtained data (dendrogram) that the 21 apricot genotypes may be divided into four groups, which have internally the same characteristics. Genotypes are structured by hierarchy, and the structuring process starts with grouping on the highest level of clustering (grouping), and then grouping goes down towards lower levels. Groups realized in these explorations display some level of similarities when genotypes have various antioxidant capacities and TP. Appliance of cluster analysis in this way is unique and very useful because it reveals groups of varieties with similar biochemical pathways. Parallel analysis based on homogenous groups points out similarities that may be useful while recommending new genotypes, because it is possible to determine similarities and also divergence from the standardized and confirmed genotypes, and in addition it is possible to assume reactions of these new varieties to production and cultivation conditions [28].

**Figure 5:**
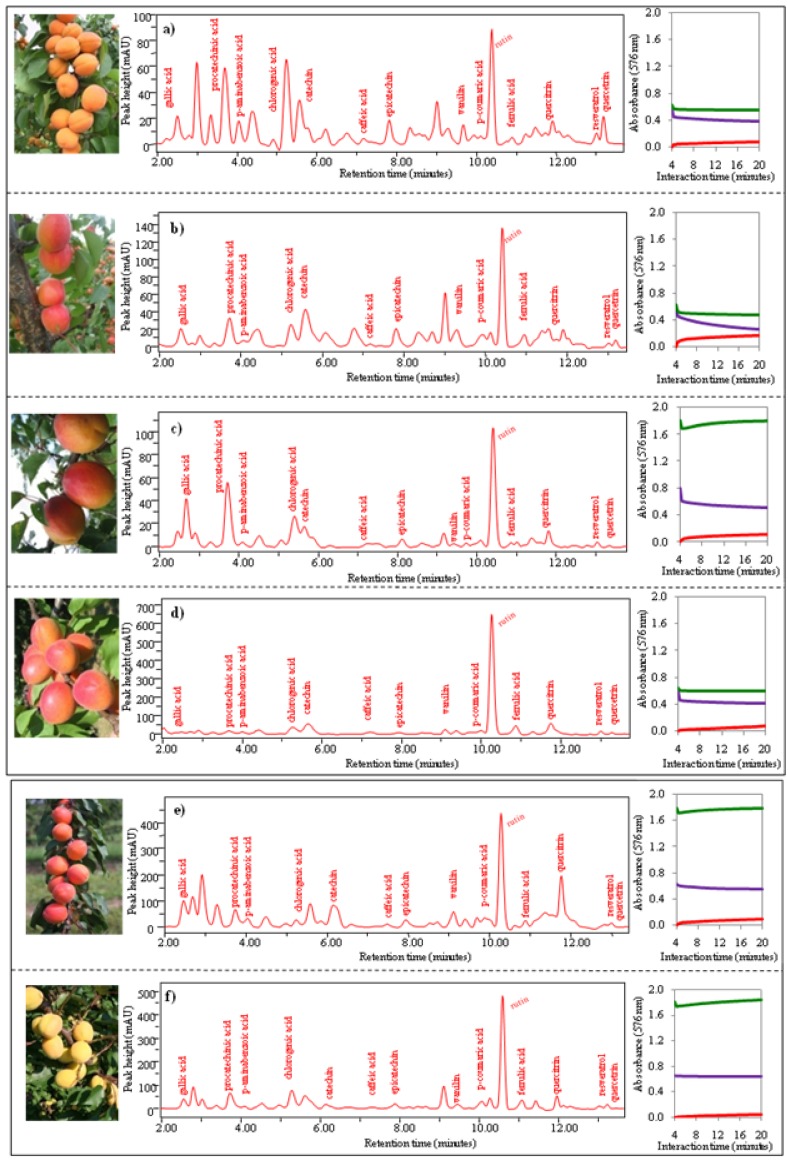
Photos of apricots, chromatograms of 15 phenolic compounds, dependence of change of absorbance of three enzyme methods (DPPH, TEAC, FRAP) on time. (**a**) GenotypeLE-1402, (**b**) LE-806, (**c**) LE-3190, (**d**) LE-985, (**e**) LE-2927 and (**f**) LE-3709.

Further, we clustered genotypes into the defined four groups according to mathematical analysis and provided the corresponding chromatograms and photos. *Group 1 *includesgenotypesLE-1402, LE-806, LE-3190, LE-985, LE-2927 and LE-3709.These genotypes demonstrated the lowest antioxidant characteristics values. This fact is probably caused by their yellow and orange tone colouring in, which suggests a low content of anthocyanins (Table 2). These compounds demonstrate high antioxidant activity and their low content is probably associated with low antioxidant activity. Photos and chromatograms are shown in Figure 5 a-f. Moreover, we show the course of change of absorbance with increasing time of interaction of the analyzed fruit extract with the specific chemicals related with the method used for determination of antioxidant activity.

**Figure 6:**
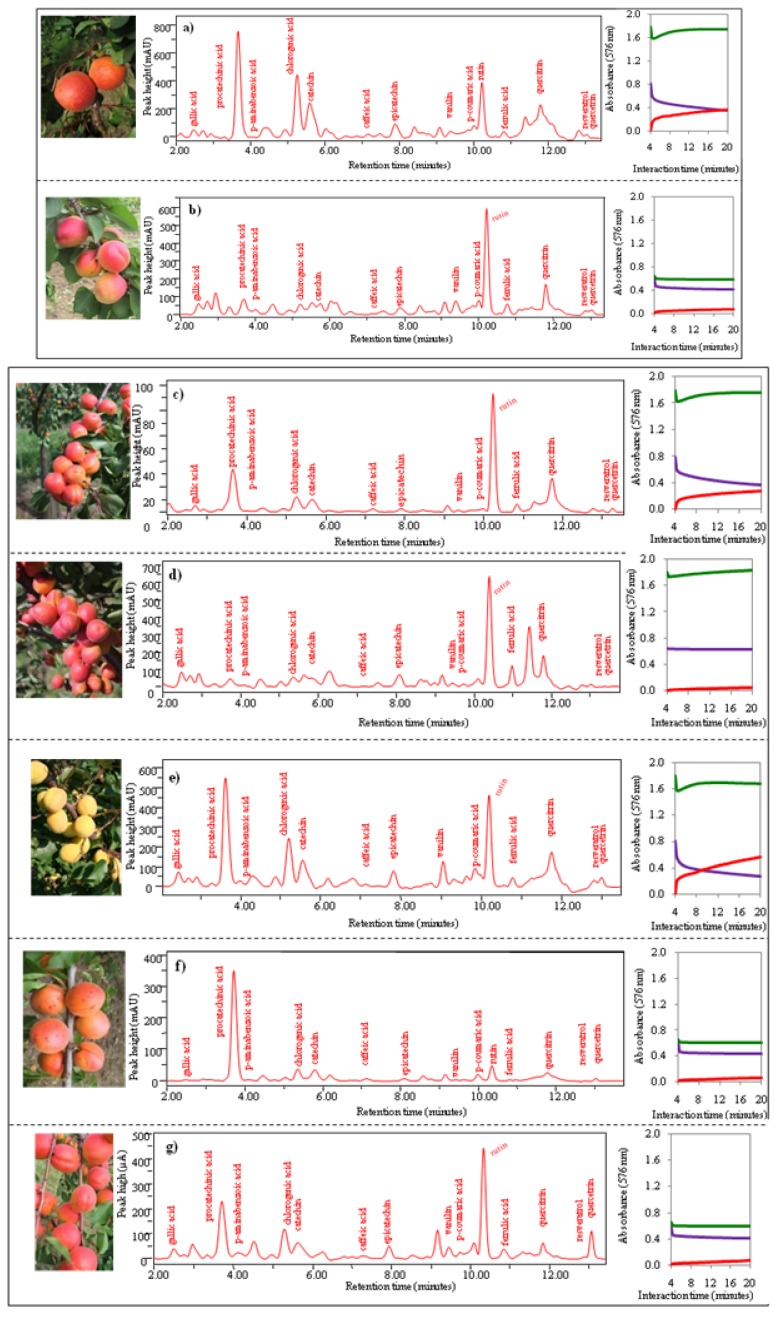
Photos of apricots, chromatograms of 15 phenolic compounds, dependence of change of absorbance of three enzyme methods (DPPH, TEAC, FRAP) on time. (**a**) Genotype LE-3239, (**b**) LE-3247, (**c**) LE-2267, (**d**) LE-10278, (**e**) LE-9299, (**f**) LE-3241 and (**g**) LE-3255.

*Group 2* includes the genotypesLE-3239, LE-3247, LE-2267, LE-10278, LE-9299, LE-3241 and LE-3255. Genotypes of this group demonstrated higher antioxidant activities according to the FRAP method (Table 1) in comparison with genotypes of other groups. Photos, chromatograms and time dependences of absorbance are shown in Figure 6a-g. It follows from the chromatographic analysis that rutin gave the highest response in all genotypes. The other similarity observed for this group was signals measured within the interval from 5 to 10 minutes. The extracts differed slightly in content of procatechin and chlorogenic acid.

**Figure 7:**
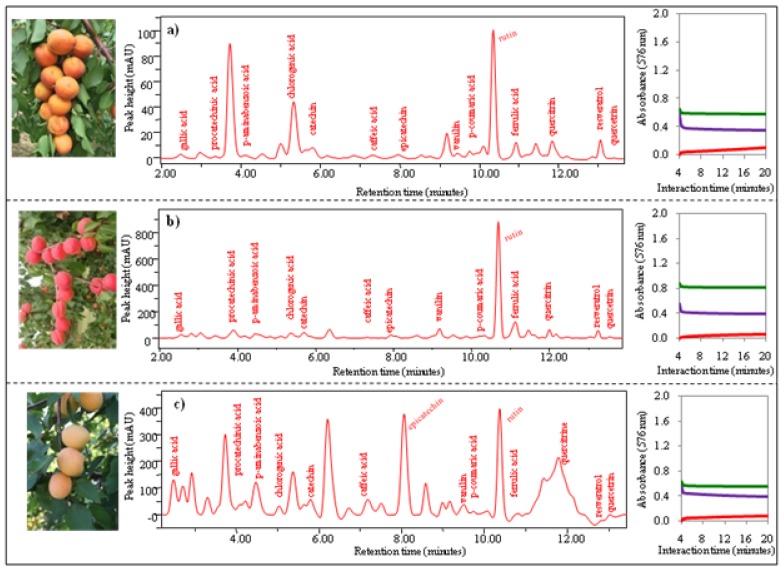
Photos of apricots, chromatograms of 15 phenolic compounds, dependence of change of absorbance of three enzyme methods (DPPH, TEAC, FRAP) on time. (**a**) GenotypeLE-8561, (**b**) LE-994 and (**c**) LE-3187.

*Group 3* includes genotypes the LE-8561, LE-994 and LE-3187. Antioxidant activity of fruits of these apricot genotypes was variable, which was closely associated with the varying content of polyphenols. Photos, chromatograms and time dependences of absorbance are shown in Figure 7 a-c. The chromatographic analysis reveals differences in this clustered group of apricot genotypes. The highest signals were measured in extract from LE-994, then LE-3187 and LE-8561. Rutin gave again the highest signal, however, procatechin differed among the genotypes.

**Figure 8:**
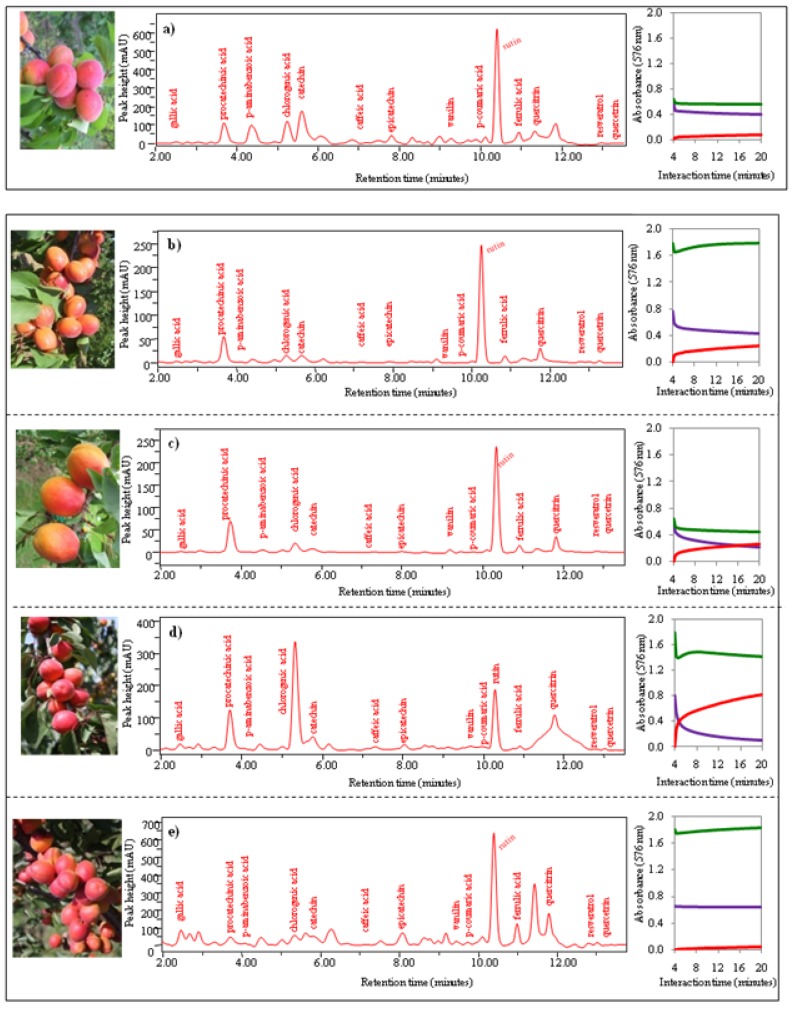
Photos of apricots, chromatograms of 15 phenolic compounds, dependence of change of absorbance of three enzyme methods (DPPH, TEAC, FRAP) on time.(**a**)GenotypeLE-3228, (**b**)LE-8175, (**c**)LE-3204, (**d**)LE-2527 and (**e**)LE-3276.

*Group *4 includes the genotypesLE-3228, LE-8175, LE-3204, LE-2527 and LE-3276. This group of these five genotypes could not be included in any group according to their antioxidant activities. Photos, chromatograms and time dependences of absorbance are shown in Figure 8a-e. Chromatographic analysis supports cluster analysis because the obtained chromatograms differed markedly according to genotype.

## 3. Experimental

### 3.1. Chemicals

All chemicals used were purchased from Sigma Aldrich (St. Louis, MO, USA) unless noted otherwise. The stock standard solutions of the reagents was prepared with ACS grade water (Sigma-Aldrich) and stored in the dark at −20 °C. Working standard solutions were prepared daily by dilution of the stock solutions with ACS water. The pH value was measured using inoLab Level 3 (Wissenschaftlich-Technische Werkstatten GmbH; Weilheim, Germany). Deionised water underwent demineralization by reverse osmosis using the instruments Aqua Osmotic 02 (Aqua Osmotic, Tisnov, Czech Republic) and then it was subsequently purified using Millipore RG (Millipore Corp., Waltham, MA, USA) – 18 MΏ MiliQ water.

### 3.2. Biological material and its basic quality parameters

Twenty one genotypes of apricot (*Prunus armeniaca *L.) were used in this study (Table 3).Plants were cultivated in Lednice, Czech Republic, climatic area T4. Fruits were collected at consumption ripeness during July and the first half of August 2009 and subsequently deep-frozen at −80 °C. 

**Table 3:** List of genotypes used in this study. Total soluble solids (TSS), titratable acidity (TA) and pH of the genotypes are given.

Genotype	TSS (◦Brix)	TA (%)	pH
LE-806	10.2	0.91	4.5
LE-985	9.1	0.98	4.8
LE-994	8.9	0.83	4.2
LE-1402	10.7	1.05	5.0
LE-2267	9.2	1.09	5.1
LE-2527	9.8	0.95	4.7
LE-2927	11.9	0.86	4.2
LE-3187	11.4	1.04	4.6
LE-3190	8.1	0.79	4.6
LE-3204	8.7	0.78	4.0
LE-3228	9.7	0.88	4.4
LE-3239	9.9	0.96	4.6
LE-3241	10.5	1.01	4.8
LE-3247	10.9	1.03	4.9
LE-3255	9.6	1.10	5.1
LE-3276	9.7	0.89	4.4
LE-3709	8.9	0.79	4.1
LE-8175	8.0	0.7	4.0
LE-8561	11.1	0.98	4.8
LE-9299	12.0	1.07	5.0
LE-10278	11.7	0.94	4.6

Chemical analyses were conducted according to Perez-Pastor *et al*. [29] and [30]. Total soluble solids (TSS) were determined in apricot extracts by using a hand refractometer (Ilabo, Czech Republic). Values were expressed as °Brix. The titratable acidity (TA) of the apricot extracts was determined by titrating 1 mL of the extract with 0.1 mol·L^-1^ NaOH and expressed as a percentage of malic acid. The pH of apricot extract was measured with an inoLab Level 3 pH-meter.

### 3.3. Sample preparation

Fruits (10 g) were transferred to mortar, deep-frozenwith liquid nitrogen and homogenized with methanol (10 mL, 99% - *w*/*w*). Homogenized samples were quantitatively transferred to test tubes and at same laboratory conditions shaken for 30 min with subsequent sonification and centrifugation (Eppendorf 5804R, Germany) for 30 min at 16,400 rpm. Supernatants were filtered through membrane discs (0.45 µm, Metachem, Torrance, CA, USA). Filtrate (500 µL) was pipetted and diluted with methanol (500 µL).

### 3.4. Spectrometric determination of antioxidant activity and total polyphenols content

Absorbance of 2 mL of solution in a cuvette (10 mm), was determined using an automated VIS spectrophotometer (BS 200, Mindray, China) at time t_0_. Reagents and samples were stored in a cooled carrousel at 4 °C and automatically pipetted into plastic cuvettes (Mindray, China) and subsequently mixed with real samples (5 μL). Changes in absorbance were measured for 21 min at 16-second intervals from mixing of reagents at below mentioned wavelengths and optical trace 5 mm. Cuvettes were incubated at 37°C. Specific experimental conditions are as follows.

#### 3.4.1. Determination of total polyphenols using Folin-Ciocalteu reagent

**Figure 9:**
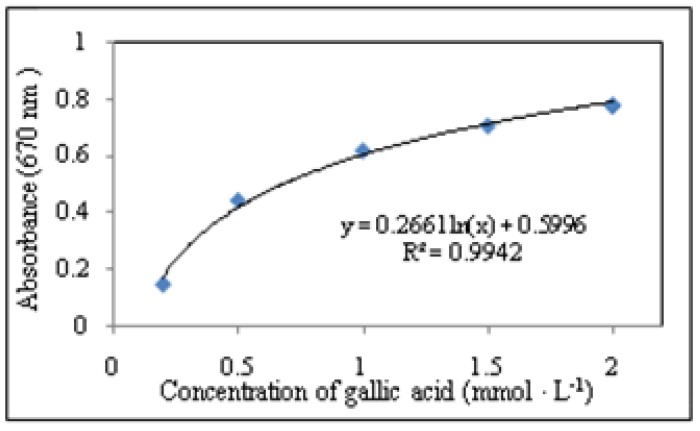
Dependence of absorbance on concentration of gallic acid within the interval from 0.25 to 2 mmol·L^-1^.

The Folin-Ciocalteu method, based on the reduction of a phosphotungsten-phosphomolybdate complex by phenolics to blue reaction products, was used for determination of phenolic compounds. Sample (0.5 mL) was pipetted into cuvette and diluted with ACS water (1.5 mL). Subsequently, Folin-Ciocalteu reagent (0.05 mL) of was added and the solution was incubated at 22 °C for 30 min., The absorbance was measured using dual-beam spectrophotometer SPECORD 210 (Carl Zeiss Jena, Germany) at wavelengths λ = 670 nm against blank (all chemicals without a sample or gallic acid) according to [14]. The absorbance was measured in triplicate. Results were expressed as equivalents of gallic acid in mg·100 g^-1^. The method was calibrated on the well known phenolic compound gallic acid. We measured logarithmic concentration dependence and used for quantification of total polyphenols content in the genotypes (Figure 9).

#### 3.4.2. Determination of antioxidant activity by DPPH test

**Figure 10:**
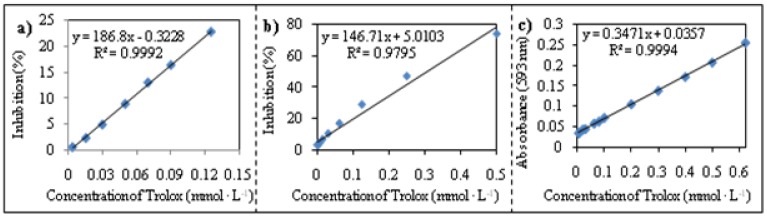
Calibration curve and equation of (**a**) DPPH test, (**b**) TEAC method and (**c**) FRAP method. Relative antioxidant activity was expressed as percentage of absorbance decrease and subsequently calculated to equivalent content of Trolox. Each sample was measured in triplicates with relative standard deviation below 2 %. Absorbance of DPPH test was measured at wavelength λ = 515 nm, TEAC at λ = 734 nm and FRAP at λ = 593 nm.

The DPPH test was performed according to Parejo *et al*. [21]. Briefly, stock solution of DPPH (1mmol·L^-1^) with absorbance (t_0_) 0.200 ± 0.01 was mixed with 0.2 M acetate buffer (1:2, *v*/*v*). Apricot extract (10 μL) was added to this solution (190 µL) and the absorbance was measured against methanol at λ = 515 nm. The decrease in the absorbance (%) was recalculated on Trolox). The calibration dependence is shown in Figure 10a.

#### 3.4.3. Determination of antioxidant activity by TEAC method

The method is based on detection of H^•^ coming from antioxidant due to scavenging of radicals according to equation: R^•^+Aox-H→RH+Aox^•^ (Aox=antioxidant). Briefly, ABTS^•^ (54.9 mg) was dissolved in 20 mL of phosphate buffer (pH 7.0; 5 mM) and activated to cation of ABTS+ radical by addition of MnO_2_ (1 g) under occasional stirring for 30 min. Solution was subsequently diluted by phosphate buffer to absorbance (t_0_) 0.500 ± 0.01. Absorbance of solution was measured at λ = 734 nm [31,32]. The calibration dependence is shown in Figure 10b.

#### 3.4.4. Determination of antioxidant activity using FRAP method

A working FRAP solution was prepared by mixing of 10 volume aliquots of acetate buffer (300 mM, pH 3.6) with 1 volume aliquot of TPTZ solution (10 mM2,4,6- tripyridyl-*s*-triazine dissolved in 40 mM HCl) and with 1 volume aliquot of FeCl_3_ solution (20 mM). Absorbance was measured at wavelength λ = 593 nm. The calibration dependence is shown in Figure 10c.

### 3.5. HPLC analysis

The HPLC-UV-VIS system consisted of two solvent delivery pumps operating within the range of 0.001–9.999 mL·min^-1^ (Model 582 ESA Inc., Chelmsford, MA, USA), Metachem Polaris C_18_A reverse-phase chromatographic column Zorbax SB C_18_ (150 × 4.6; size of particles 5 µm, Agilent Technologies, Palo Alto, CA, USA), UV detector Shimadzu (Model 528, ESA, USA). Both the detector and the reaction coil/column were thermostated. The sample (15 μL) was injected using autosampler (Model 540 Microtiter HPLC, ESA, USA). Chromatographic conditions were optimized accordingly, flow rate of mobile phase 1 mL·min^-1^, temperature 30 °C. Isocratic mobile phase was as follows: A: acetic acid (50 mM) and B: acetic acid (50 mM) in acetonitrile. Spectrometric detector scanned responses at 260 nm.

### 3.6. Mathematical and statistical analysis

Mathematical and statistical analysis of experimental data was carried out in package MATLAB®, Version 7.9.0.529 (R2009b, MathWorks Inc., Natick, MA, USA). Ward's error sum of squares method was used for calculations. Choice of this method was very advantageous, especially in our case, where all parameters are of identical measure. The method works on principle of connection of two similar objects into clump on basis of “criterion of analysis quality into sum of squares of objects deviations from vector of average of correspondent cluster“. At the beginning, there is matrix of vectors of three measured values for all apricot genotypes = objects, from which matrix of distances between all couples of objects on basis of Euclidean distance in accordance with thereinafter formula is calculated:

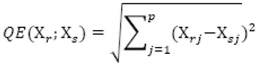

where x_r,s_ are two measured vectors of corresponding objects and j indicates element of vector (p = 3). Matrix of distances is minimized by method of connection of objects into clusters; reciprocally similar objects from matrix are chosen in accordance of minimal distances. Vector of their averages is subsequently calculated, which means node of their connection as minimum of sum of deviations squares from clustering average. Simplified arithmetical relation for determination of sum of squares in both evanescent clusters is given as:

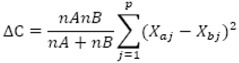


Generally, it is arithmetic product of Euclidean distance between nodes of given clusters A and B connected into one new cluster C, which depends on number of objects in given clusters n_A,B_. In each step, connection of minimizations of criterion ΔC is implemented; method itself tends to form small clusters of the same sizes, which appears to be optimal.

## 4. Conclusions

Human health and nutrition are still one of the most studied and interesting topics. Natural compounds, including those coming from plants, are nowadays under detailed investigation due to their potentially beneficial effects. In this study, we aimed at characterization of various genotypes of apricots resistant against PPV. The apricot fruits were analyzed from various analytical points of view including antioxidant capacities, total polyphenols content and identification and determination of fifteen phenols. To find similarities among the genotypes, cluster analysis of the data obtained was used. It can be concluded that some genotypes are similar from the biochemical point of view, which could be of interest, not only from the nutritional point of view, but also from the point of view of breeding of apricots.
